# Renal Sinus Fat Invasion and Tumoral Thrombosis of the Inferior Vena Cava-Renal Vein: Only Confined to Renal Cell Carcinoma

**DOI:** 10.1155/2014/140365

**Published:** 2014-11-18

**Authors:** Turker Acar, Mustafa Harman, Serkan Guneyli, Sait Sen, Nevra Elmas

**Affiliations:** ^1^Department of Radiology, Mevlana University School of Medicine, Yeni Istanbul Caddesi No. 235, Selcuklu, 42003 Konya, Turkey; ^2^Department of Radiology, Ege University School of Medicine, Bornova, 35100 Izmir, Turkey; ^3^Department of Radiology, Bulent Ecevit University School of Medicine, 67600 Zonguldak, Turkey; ^4^Department of Pathology, Ege University School of Medicine, Bornova, 35100 Izmir, Turkey

## Abstract

Epithelioid angiomyolipoma (E-AML), accounting for 8% of renal angiomyolipoma, is usually associated with tuberous sclerosis (TS) and demonstrates aggressive behavior. E-AML is macroscopically seen as a large infiltrative necrotic tumor with occasional extension into renal vein and/or inferior vena cava. However, without history of TS, renal sinus and venous invasion E-AML would be a challenging diagnosis, which may lead radiologists to misinterpret it as a renal cell carcinoma (RCC). In this case presentation, we aimed to report cross-sectional imaging findings of two cases diagnosed as E-AML and pathological correlation of these aforementioned masses mimicking RCC.

## 1. Introduction

Renal cell carcinoma (RCC) is the most common adult renal epithelial cancer that covers more than 90% of all renal malignancies [1]. According to American Cancer Society statistics, about 63,920 new cases of kidney cancer will occur and approximately 13,860 people will die from this disease in 2014 [2]. Cross-sectional imaging modalities, such as computed tomography (CT) and magnetic resonance imaging (MRI) in RCC, are critical since they confirm a suspected renal mass by enhancement pattern, allow distinction between solid and cystic lesions, give information about involvement of regional lymph nodes at the time of diagnosis, and are also beneficial in demonstrating distant metastasis. Clear cell RCC, the most common histological subtype, accounts for 70% of all RCCs [1]. Due to hypervascularity caused by vascular and growth factors resulting from tumor suppressor genes inactivation, clear cell RCCs typically show enhancement on contrast-enhanced studies [3]. Renal vein (RV) and/or inferior vena cava (IVC) invasion is a common finding that can be seen up to 45% of RCCs [4].

Except for RV and/or IVC invasion, renal sinus fat invasion as a prognostic significance can be also seen in a RCC [5]. These two aforementioned imaging features are typically confined to malignant kidney masses, but venous and renal sinus fat invasion can be rarely seen in epithelioid angiomyolipoma (E-AML), which is a benign mesenchymal tumor of the kidney. In this case presentation we aimed to report imaging features of 2 distinct cases, who were diagnosed as E-AML resembling RCC on cross-sectional imaging. Written informed consents were obtained from each patient.

## 2. Case Presentation

### 2.1. Case 1

**Figure 1:**
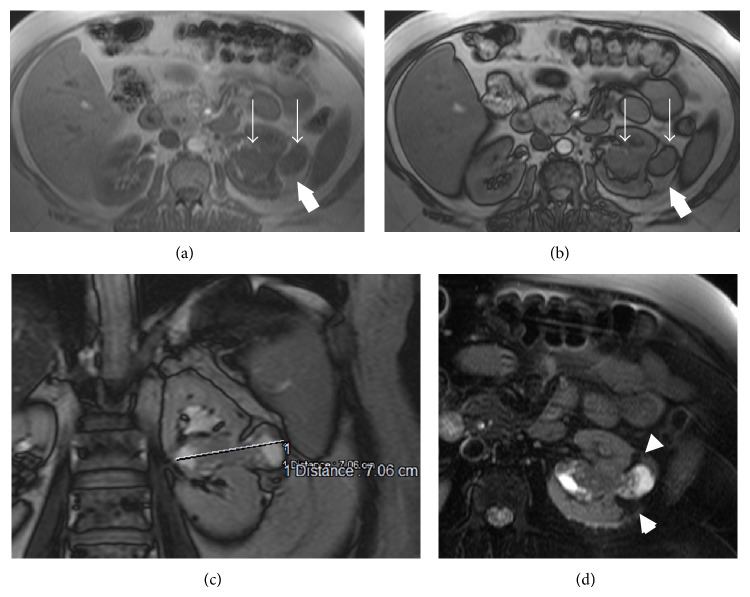
(a–d) E-AML both contains fatty and nonadipose components. T1W axial in-phase (a) and out-phase images (b) reveal mass lesion involving renal sinus and subcapsular region. No apparent fat is seen in some portion (thin arrows). However, due to pure fat content of subcapsular tumor component, no signal reduction is seen in dual echo imaging (thick arrow). TruFISP coronal image (c) shows subcapsular extension and maximum diameter. Axial T2W fat-saturated image (d) shows solid infiltrative renal mass with renal sinus and pelvicalyceal extension; note that pure fatty portion of subcapsular component demonstrates signal reduction (arrowheads).

**Figure 2:**
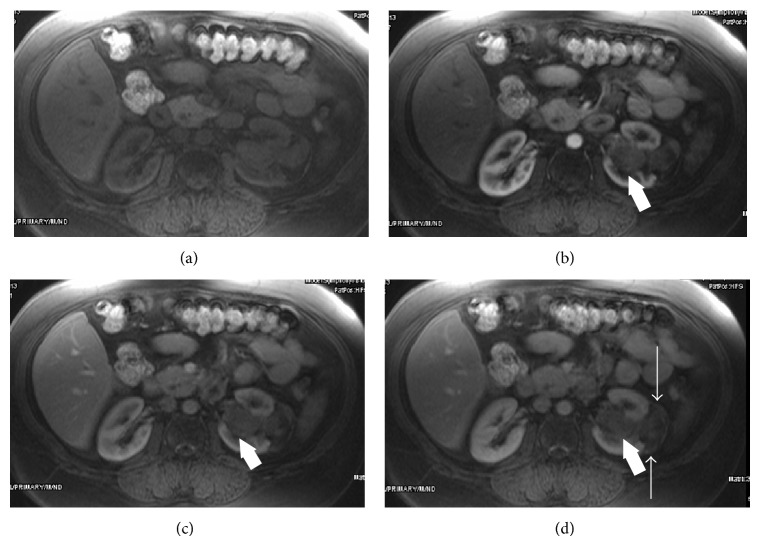
(a–d) Precontrast axial T1W fat-saturated (a), early arterial (b), late arterial (c), and portal venous phase (d) images show slightly increased enhancement in fatty areas of subcapsular portion in portal phase (thin arrow). However, nonadipose component of E-AML is not avidly enhancing in the postcontrast images (thick arrows).

**Figure 3:**
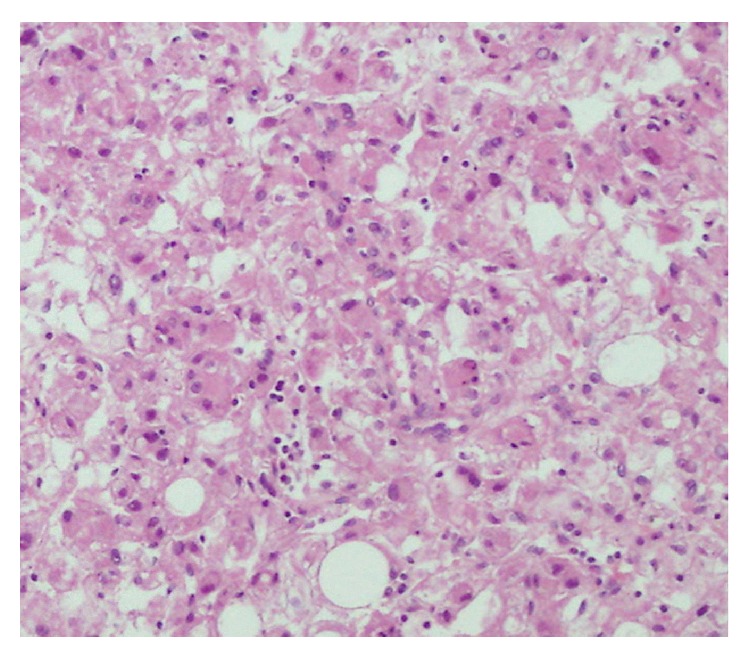
Hematoxylin-eosin staining of the same lesion is depicted. Large epithelioid cells show pleomorphism with large hyperchromatic nuclei and abundant eosinophilic cytoplasm in epithelioid angiomyolipoma are demonstrated. Same image also contains fatty tissue that corresponds with subcapsular adipose component of the E-AML.

A 53-year-old woman with left flank pain that started 1 year before was admitted to urology department of our institution. Her physical examination was normal except for left-sided costovertebral angle tenderness. Complete blood count and blood biochemistry tests were in normal limits, but microscopic hematuria was detected in urinalysis. She initially underwent ultrasonography with the preliminary diagnosis of kidney stone. Upon detection of a solid mass at the mid-portion of left kidney, a CT examination was planned, but she underwent MRI because of iodinated contrast media allergy that she experienced before. On MRI, a 7 cm kidney mass with infiltrative characteristics, originating from central part of the left kidney with the involvement of the pelvicalyceal system, was revealed (Figures 1 and 2). Following imaging workup, a radical nephrectomy was performed. The specimens were proved to be E-AML upon detection of large epithelioid cells (Figure 3) as well as positive immunohistochemical stains with melanosome associated proteins (HMB-45 and Melan-A).

### 2.2. Case 2

**Figure 4:**
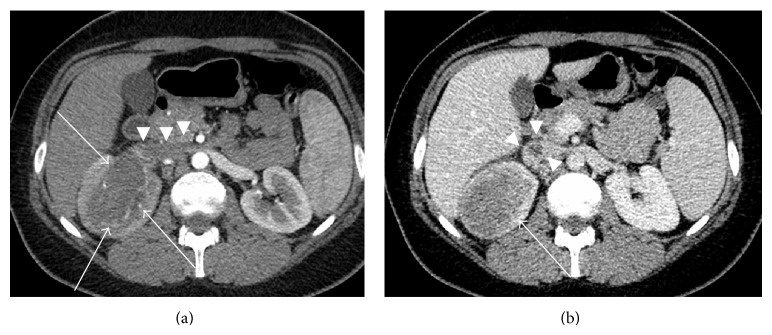
(a, b) Axial venous phase postcontrast CT images (a, b) from the mid-portion of the kidneys. There is a hypoattenuating mass lesion involving the right central part of the kidney with sinus extension (arrows). Hypodense thrombus materials are seen in both right renal vein and inferior vena cava (arrowheads).

A 30-year-old woman with recurrent right-sided fullness that started 6 months ago was admitted to internal medicine department of our institution. Neither physical examination nor blood test was remarkable. She was referred to radiology department for ultrasonography. Right kidney enlargement and a solid mass with heterogeneous echogenicity were detected during ultrasonography. Upon detection of right-sided kidney mass, a biphasic CT scan was performed. CT revealed a kidney mass measuring 8.5 cm with involvement of RV and IVC at the central part of the right kidney (Figure 4). Following imaging workup, she underwent radical nephrectomy with the presumed diagnosis of RCC, but the pathology report was consistent with E-AML similar to the first case.

## 3. Discussion

Angiomyolipoma (AML), constituting %1 of all surgically removed kidney masses, is the most common benign mesenchymal neoplasm of the kidney. AML is composed of variable degree of mature adipose tissue, smooth muscle, and blood vessels [1]. Two histological subtypes of renal AML are described in the literature; the first is the triphasic (classic) variant and the second is epithelioid [6, 7]. These two aforementioned subtypes show variable biological behavior, which gives distinctive cross-sectional imaging findings. A classic AML of the kidney is seen as a hyperechoic mass on ultrasonography due to the macroscopic fat, hypoattenuating lesion on CT, and hypointense lesion on fat-saturated pulse sequences of MRI [8, 9].

On the other hand, E-AML (pathologically known to be as perivascular epithelioid cell tumors) which accounts for 8% of renal angiomyolipomas, is usually associated with tuberous sclerosis (TS) and demonstrates aggressive behavior [10]. This extremely rare type of angiomyolipoma was first described by Eble et al. [11] in 1997. No gender predilection was described and the median age is 38 years in E-AML [1]. Pathologically, E-AML demonstrates layers of epithelioid cells with nuclear pleomorphism and mitoses ranging from oval to polygonal in shape. At immunohistochemical analysis, E-AML shows coexpression of melanocytic markers such as HMB-45 and Melan-A [10] helping in differentiation from RCC. Another immunohistochemical feature of E-AML is the lack of epithelial membrane antigen and cytokeratin, which is typically positive in RCC [1].

E-AMLs are regarded as potentially malignant lesions and as the tumor enlarges, the more likely it will spread [12]. Nese et al. [13] conducted a clinicopathological study of 41 patients, who were diagnosed to be as pure E-AML. According to the aforementioned study, only 9 patients had TS history, whereas the remaining patients had not. Ratios of tumor recurrence and metastasis were 17% and 49%, respectively. Lymphatic spread was detected in 24% of the patients [13]. In accordance with the literature, our two patients did not have TS history, which challenged the diagnosis. Regarding the malignant potential of E-AML as stated in the literature, in addition to abdominal imaging thorax CT was also performed for each patient, which was negative for metastatic disease. In addition to the malignant potential, E-AMLs may have varying degree of fat cells. Froemming et al. [14] found that small foci of fat can be detected with CT or MRI in some patients diagnosed with E-AML. In the current study we have also seen small amount of fat in the subcapsular region of first case.

On imaging, E-AML is seen as a large mass, which may contain hemorrhage and necrosis. E-AML is even larger than fat poor AML with a mean diameter of 7 cm. Small amount of fat can be seen on CT and/or MRI. Contrary to classic AML, E-AML is prevalently seen as a hyperattenuating lesion on unenhanced CT examination and it is presented as a hypointense lesion on T2-weighted images due to epithelioid muscle ingredient [14, 15]. Tsukada et al. [15] reported that E-AML reveals a variable appearance including heterogeneously or homogeneously enhancing solid mass or as multilocular cystic mass which may lead misinterpretation as a RCC. Another radiological feature that leads to difficulty in distinction of E-AML from RCC is that the former tumor may also demonstrate extension into RV or IVC just as the latter one [8]. In the current study, in accordance with the literature, E-AMLs were detected as large masses. The MRI workup of the first E-AML revealed decreased T2-weighted signal intensity due to epithelioid muscle components, necrotic portions, and small amount of fat of the extrarenal component. CT workup of the second E-AML presented as a large heterogeneously enhancing mass with venous spread, which may be misdiagnosed as RCC.

There are three major subtypes of RCC: clear cell (70%), papillary (10%), and chromophobe (5%). Clear cell RCCs are typically seen as hypervascular tumors avidly enhancing on contrast-enhanced studies including CT, MRI, and angiography. On the other hand, papillary RCCs appear as hypovascular and homogeneous on CT and MRI. Another interesting feature of papillary RCC is that it may rarely contain macroscopic fat [16]. Chromophobe RCCs show relatively homogeneous enhancement at CT and MRI and they may demonstrate spoke-wheel pattern of contrast enhancement which was also described for renal oncocytoma [16]. Unlike clear cell RCC, E-AMLs in our series demonstrated insignificant contrast enhancement. In the current study, E-AMLs were seen as hypovascular tumoral lesion in the first case, and as slight heterogeneous enhancing mass lesion in the second case. Moreover, subcapsular tumor component of the first E-AML contained some degree of adipose tissue which may also be seen in papillary RCC. In summary, E-AMLs in our series revealed varying degrees of radiologic similarities with papillary and chromophobe RCC.

Although the detection of fat is a well-known imaging feature of classic AML, an important diagnostic issue is that some rare types of AMLs may contain few or no fatty tissue (e.g., fat poor AML). Since malignancies can also demonstrate these imaging characteristics [17], informing the pathologist and sharing detailed imaging findings before final diagnosis is critical.

Surgical resection is the main treatment option of E-AML. However, mammalian target of rapamycin (mTOR) inhibitors, such as temsirolimus or sirolimus, are reported to be medical management option for E-AML [18, 19].

In conclusion, lack of macroscopic fat, renal sinus invasion, and RV-IVC extension and without given the history of TS, E-AML is a challenging diagnosis on cross-sectional imaging. If pathological and immunohistochemical analysis confirm this diagnosis, a full body scan is necessary because of the metastatic potential of this rare type of AML.

## References

[1] Eble J. N., Sauter G., Epstein J. I., Sesterhenn I. A. (2004). *World Health Organization Classification of Tumors: Pathology and Genetics. Tumors of the Urinary System and Male Genital Organs*.

[2] American Cancer Society Kidney Cancer (Adult)—Renal Cell Carcinoma. http://www.cancer.org/cancer/kidneycancer/detailedguide/kidney-cancer-adult-key-statistics.

[3] Cohen H. T., McGovern F. J. (2005). Renal-cell carcinoma. *The New England Journal of Medicine*.

[4] Moch H., Gasser T., Amin M. B., Torhorst J., Sauter G., Mihatsch M. J. (2000). Prognostic utility of the recently recommended histologic classification and revised TNM staging system of renal cell carcinoma: a Swiss experience with 588 tumors. *Cancer*.

[5] Kim C., Choi H. J., Cho K.-S. (2014). Diagnostic value of multidetector computed tomography for renal sinus fat invasion in renal cell carcinoma patients. *European Journal of Radiology*.

[6] Tamboli P., Ro J. Y., Amin M. B., Ligato S., Ayala A. G. (2000). Benign tumors and tumor-like lesions of the adult kidney part II: benign mesenchymal and mixed neoplasms, and tumor-like lesions. *Advances in Anatomic Pathology*.

[7] Prasad S. R., Sahani D. V., Mino-Kenudson M., Narra V. R., Humphrey P. A., Menias C. O., Chintapalli K. N. (2007). Neoplasms of the perivascular epithelioid cell involving the abdomen and the pelvis: Cross-sectional imaging findings. *Journal of Computer Assisted Tomography*.

[8] Katabathina V. S., Vikram R., Nagar A. M., Tamboli P., Menias C. O., Prasad S. R. (2010). Mesenchymal neoplasms of the kidney in adults: imaging spectrum with radiologic-pathologic correlation. *Radiographics*.

[9] Siegel C. L., Middleton W. D., Teefey S. A., McClennan B. L. (1996). Angiomyolipoma and renal cell carcinoma: US differentiation. *Radiology*.

[10] Aydin H., Magi-Galluzzi C., Lane B. R., Sercia L., Lopez J. I., Rini B. I., Zhou M. (2009). Renal angiomyolipoma: Cclinicopathologic study of 194 cases with emphasis on the epithelioid histology and tuberous sclerosis association. *The American Journal of Surgical Pathology*.

[11] Eble J. N., Amin M. B., Young R. H. (1997). Epithelioid angiomyolipoma of the kidney: a report of five cases with a prominent and diagnostically confusing epithelioid smooth muscle component. *American Journal of Surgical Pathology*.

[12] Tsai C.-C., Wu W.-J., Li C.-C., Wang C.-J., Huang C.-H., Wu C.-C. (2009). Epithelioid angiomyolipoma of the kidney mimicking renal cell carcinoma: a clinicopathologic analysis of cases and literature review. *Kaohsiung Journal of Medical Sciences*.

[13] Nese N., Martignoni G., Fletcher C. D., Gupta R., Pan C.-C., Kim H., Ro J. Y., Hwang I. S., Sato K., Bonetti F., Pea M., Amin M. B., Hes O., Svec A., Kida M., Vankalakunti M., Berel D., Rogatko A., Gown A. M. (2011). Pure epithelioid PEComas (so-called epithelioid angiomyolipoma) of the kidney: a clinicopathologic study of 41 cases: detailed assessment of morphology and risk stratification. *American Journal of Surgical Pathology*.

[14] Froemming A. T., Boland J., Cheville J., Takahashi N., Kawashima A. (2013). Renal epithelioid angiomyolipoma: imaging characteristics in nine cases with radiologic-pathologic correlation and review of the literature. *The American Journal of Roentgenology*.

[15] Tsukada J., Jinzaki M., Yao M. (2013). Epithelioid angiomyolipoma of the kidney: radiological imaging. *International Journal of Urology*.

[16] Prasad S. R., Humphrey P. A., Catena J. R., Narra V. R., Srigley J. R., Cortez A. D., Dalrymple N. C., Chintapalli K. N. (2006). Common and uncommon histologic subtypes of renal cell carcinoma: imaging spectrum with pathologic correlation. *Radiographics*.

[17] Jinzaki M., Silverman S. G., Akita H., Nagashima Y., Mikami S., Oya M. (2014). Renal angiomyolipoma: a radiological classification and update on recent developments in diagnosis and management. *Abdominal Imaging*.

[18] Wolff N., Kabbani W., Bradley T., Raj G., Watumull L., Brugarolas J. (2010). Sirolimus and temsirolimus for epithelioid angiomyolipoma. *Journal of Clinical Oncology*.

[19] Shitara K., Yatabe Y., Mizota A., Sano T., Nimura Y., Muro K. (2011). Dramatic tumor response to everolimus for malignant epithelioid angiomyolipoma. *Japanese Journal of Clinical Oncology*.

